# Double filtration plasmapheresis in antithyroid drug-refractory Graves disease with a massive goiter

**DOI:** 10.1210/jcemcr/luag095

**Published:** 2026-04-28

**Authors:** Miku Yamazaki, Hiroyuki Uchinuma, Yuka Yokota, Hiroki Ishii, Takeshi Moriguchi, Kyoichiro Tsuchiya

**Affiliations:** Department of Diabetes and Endocrinology, University of Yamanashi Hospital, Yamanashi 409-3898, Japan; Department of Diabetes and Endocrinology, University of Yamanashi Hospital, Yamanashi 409-3898, Japan; Department of Pathology, University of Yamanashi Hospital, Yamanashi 409-3898, Japan; Department of Otolaryngology, Head and Neck Surgery, University of Yamanashi, Yamanashi 409-3898, Japan; Department of Emergency and Critical Care Medicine, University of Yamanashi Hospital, Yamanashi 409-3898, Japan; Department of Diabetes and Endocrinology, University of Yamanashi Hospital, Yamanashi 409-3898, Japan

**Keywords:** Graves disease, ATD-resistant thyrotoxicosis, DFPP, large goiter, total thyroidectomy

## Abstract

We report a case of double filtration plasmapheresis (DFPP) performed in medication-resistant Graves disease (GD) with a massive goiter. A 19-year-old female was diagnosed with GD and initially demonstrated poor medication compliance. She presented with loss of consciousness from hypoxemia due to goiter-related tracheal narrowing. Despite inpatient drug therapy, the thyroid hormone levels remained high. We performed DFPP to control thyrotoxicosis before proceeding with thyroidectomy. After 4 sessions, thyroid hormone levels improved, enabling total thyroidectomy. GD with a large goiter may be refractory to medical therapy. In such cases, DFPP can improve thyroid hormone levels.

## Introduction

The first-line treatment options for Graves disease (GD) are antithyroid drugs (ATDs), surgical therapy, and radioiodine (^131^I) therapy. In Japan, methimazole (MMI) is recommended as the first-choice ATD, except in early pregnancy [1]. However, some patients develop ATD-resistant thyrotoxicosis, characterized by persistent hyperthyroidism despite maximal antithyroid medication. Although therapeutic resistance is often associated with thyroid enlargement, clinically significant tracheal compression in GD is uncommon, with benign thyroid disease accounting for 1-2% of tracheal stenosis and life-threatening airway compromise in <1% of cases [2]. The presence of a massive goiter not only complicates medical management but also poses potential risks to patient safety, necessitating careful therapeutic consideration. While potassium iodide (KI), lithium carbonate, cholestyramine, and glucocorticoids have been reported as treatment options for ATD-resistant cases, the role of plasma exchange (PE) remains unclear, despite its established role in thyroid storm. Double filtration plasmapheresis (DFPP), a form of therapeutic PE, reduces pathogenic antibodies with less coagulation factor loss than conventional PE. We report a case demonstrating DFPP as a preoperative bridge to surgery in ATD-resistant GD with a massive goiter.

## Case presentation

A 19-year-old female presented to her local physician in September 2023 with symptoms of thyrotoxicosis including palpitations, sweating, and weight loss. Laboratory evaluation upon referral to our hospital in October 2023 revealed severe hyperthyroidism with thyroid-stimulating hormone (TSH) < 0.005 μIU/mL (reference range [RR]: 0.35-4.94 μIU/mL), free thyroxine (FT4) > 7.77 ng/dL (>100.02 pmol/L) (RR: 0.70-1.48 ng/mL; 9.01-19.05 pmol/L), free triiodothyronine (FT3) 30.20 pg/mL (46.40 pmol/L) (RR: 1.71-3.71 ng/mL; 2.63-5.70 pmol/L), and markedly elevated TSH receptor antibody (TRAb) at 24.6 IU/L (RR: <1.22 IU/L). On arrival, her blood pressure (BP) was 135/75 mmHg with a heart rate (HR) of 117 beats/min. She had a massive, non-tender World Health Organization (WHO) II goiter. Ultrasonography demonstrated bilateral thyroid enlargement with increased vascularity but no nodules: the right lobe measured 3.0 × 3.1 cm and the left lobe 2.7 × 3.2 cm (anterior–posterior × transverse), consistent with GD. Considering her age, body weight, and the need to minimize the risk of adverse effects, treatment with MMI (15 mg/day) and KI (50 mg/day) was initiated [1]. However, the therapeutic response was compromised by poor medication compliance acknowledged by the patient.

**Figure 1:**
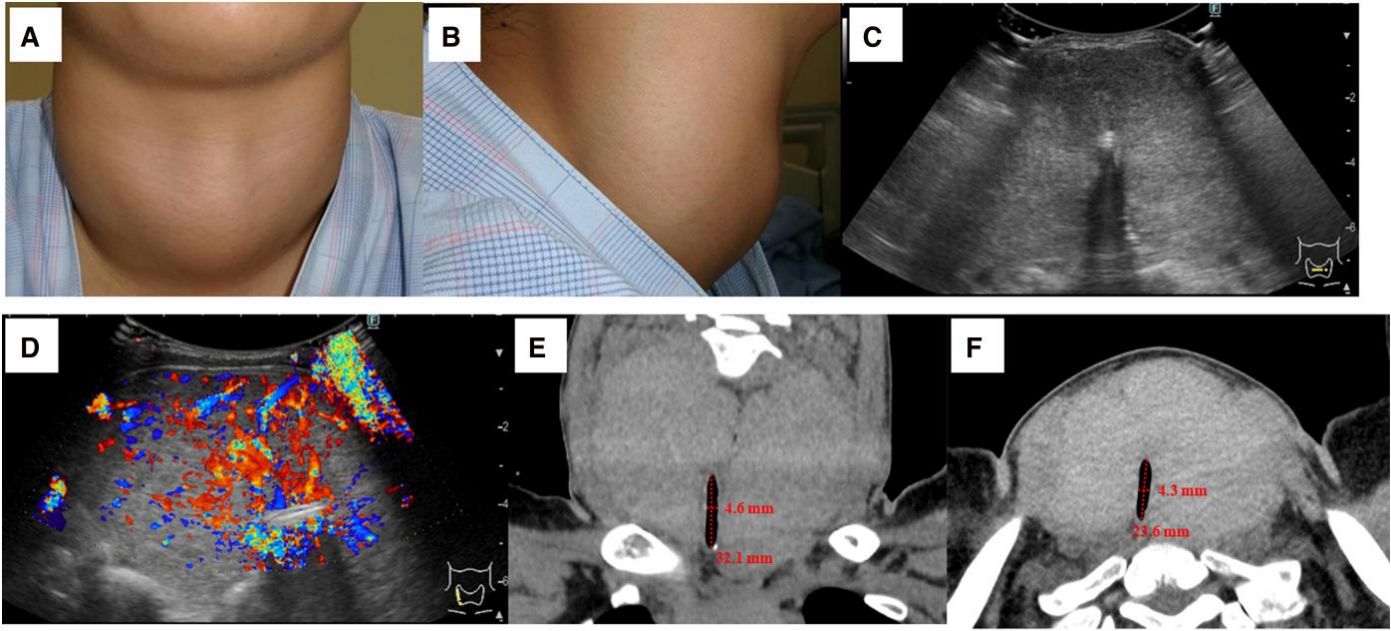
Imaging of the massive goiter. (A, B) Physical examination revealed a large goiter of WHO Classification Grade III with no tenderness or palpable mass. (C, D) Ultrasonography revealed marked thyroid enlargement with diffuse hypervascularity. The right lobe measured 5.7 × 5.2 × 8.8 cm and the left lobe 5.2 × 4.9 × 10.0 cm (anterior–posterior × transverse × longitudinal). No hypoechoic areas or masses were observed. (E-F): CT scan showing goiter (volume: 366 mL) associated with tracheal compression and narrowing with a minimal diameter of 4.3 mm.

In July 2024, she presented to our hospital with acute loss of consciousness. Vital signs were as follows: BP, 132/87 mmHg; HR, 118 beats/min; respiratory rate 22/min, and body temperature 36.9 °C. Despite requiring 6L of oxygen support during ambulance transport, her oxygen saturation improved to 97% on room air after arrival, with recovery of consciousness to Glasgow Coma Scale E4V4M6. Physical examination revealed a massive goiter classified as WHO Grade III (Fig. 1A-1B). She had intermittent cough for several days. Based on the Burch–Wartofsky score, she did not meet the diagnostic criteria for thyroid storm.

## Diagnostic assessment

Ultrasonography revealed marked thyroid enlargement with diffuse hypervascularity. The right lobe measured 5.7 × 5.2 × 8.8 cm and the left lobe 5.2 × 4.9 × 10.0 cm (anterior–posterior × transverse × longitudinal; Fig. 1C, 1D). Chest radiography showed marked tracheal narrowing without pulmonary infiltrates, and computed tomography confirmed a 366 mL thyroid compressing the trachea to 4.3 mm (Fig. 1E, 1F). Laboratory studies showed persistent thyrotoxicosis with TSH <0.005 μIU/mL, FT4 4.63 ng/dL (59.60 pmol/L), FT3 24.5 pg/mL (37.63 pmol/L), and elevated thyroid autoantibodies (TRAb) 78.1 IU/L, thyroid stimulating antibody (TSAb) 4210% [RR: <130%]). Electrocardiogram showed sinus tachycardia. Intermittent MMI and KI use likely partially suppressed thyroid hormone synthesis, improving FT4 and FT3 from initial presentation. The progressive thyroid enlargement was considered to result from chronic stimulation by TSAb. Transient loss of consciousness from tracheal stenosis resolved without persistent hypoxia; tracheostomy was reserved.

## Treatment

**Figure 2:**
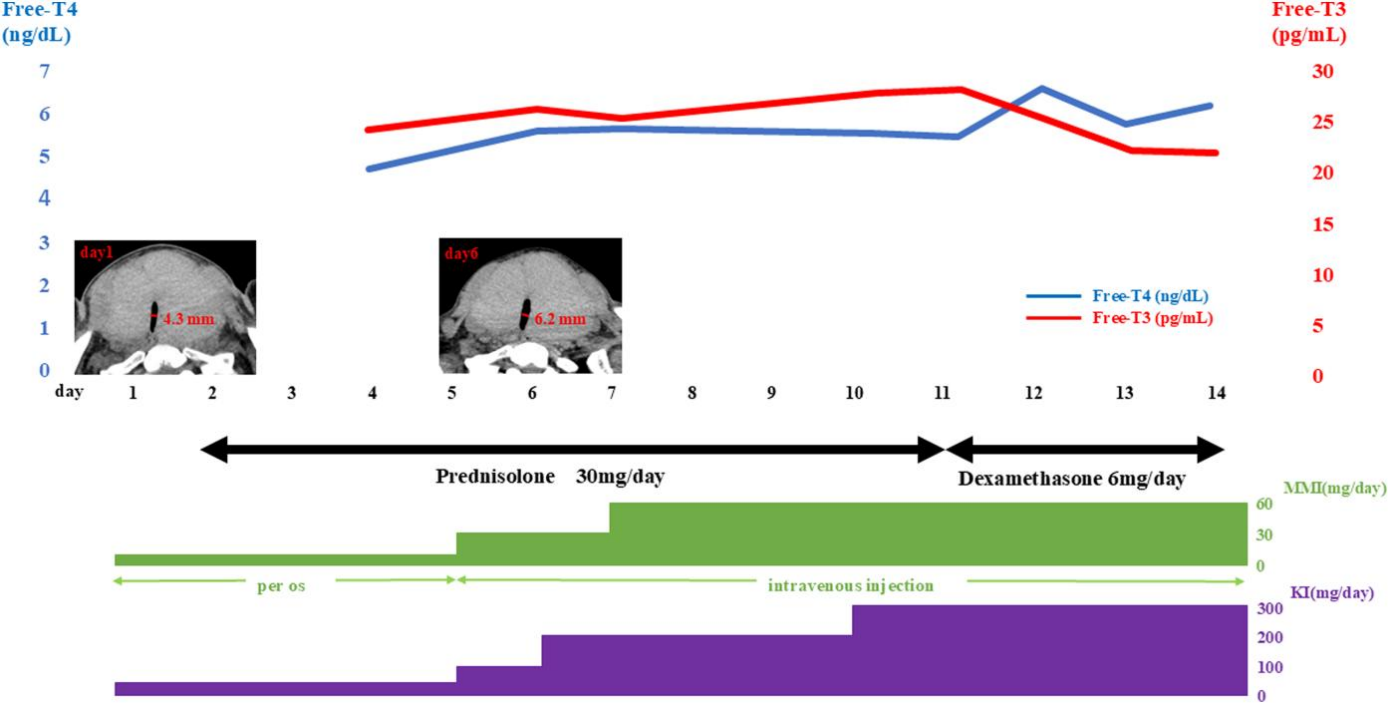
Medical stabilization before surgical intervention is necessary. We increased the dosage of MMI, KI, and dexamethasone to 60 mg/day, 300 mg/day, and 6 mg/day, respectively, from 15 mg/day, 50 mg/day, and 30 mg/day, respectively; however, thyroid hormone levels remained high. A repeat CT scan on day 9 showed improvement, with a minimal airway diameter of 6 mm; however, stenosis associated with thyroid gland enlargement persisted.

Owing to respiratory risks, we pursued medical stabilization before thyroidectomy. In patients with severe thyroid storm, a high initial dose of methimazole (60 mg/day) may be considered. However, high doses of antithyroid drugs are associated with an increased frequency of adverse effects [3]. For the same reasons as at initiation, we started intravenous injection of MMI 15 mg/day, prednisolone 30 mg/day, and oral administration of KI 50 mg/day but the thyroid hormone levels remained elevated, requiring escalation to MMI 60 mg/day, dexamethasone 6 mg/day, and KI 300 mg/day (Fig. 2). Despite triple therapy with high-dose MMI, KI, and corticosteroids, thyroid hormone levels remained elevated and although follow-up imaging demonstrated improvement in tracheal diameter from 4.3 mm to 6.2 mm, airway stenosis remained (Fig. 2).

**Figure 3:**
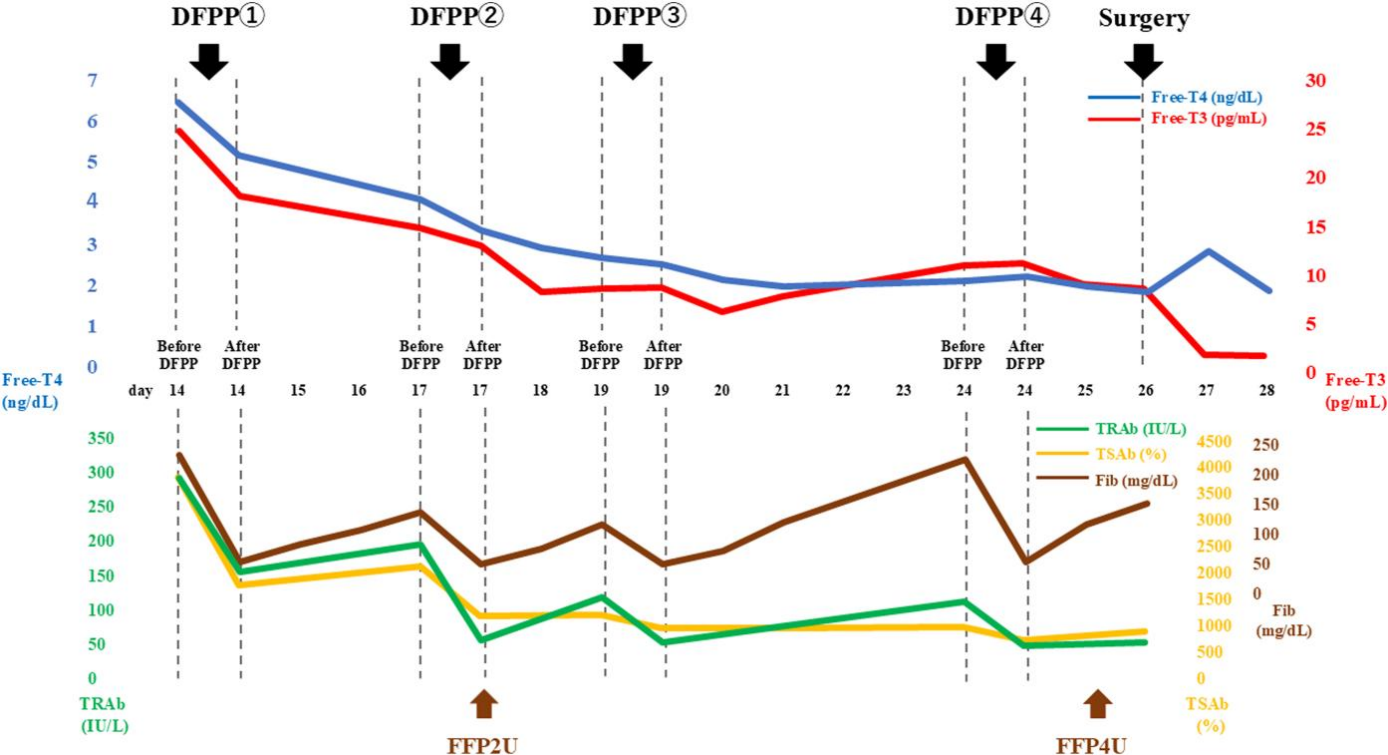
Course of treatment with DFPP. DFPP was perfomed on admission days 14, 17, 19, and 24. After 4 DFPP sessions, laboratory improvement was noted with FT4:1.94 ng/dL, FT3: 8.47 pg/mL, TRAb: 35.4 IU/L on day 26, the day of the surgery. Two and 4 units of FFP were administered on days 17 and 25, respectively.

Given refractory thyrotoxicosis and urgent surgical need, we initiated DFPP on hospital days 14, 17, 19, and 24 (Fig. 3). Two filters were used: a plasma separator and a plasma component separator. The target volume of 2.6 L was processed at a blood flow rate of 100 mL/min, using 12.1% human albumin solution for volume replacement.

The first DFPP session significantly improved thyroid function parameters (Table 1) while decreasing fibrinogen from 226 mg/dL (6.7 µmol/L) (RR: 200-393 mg/dL; 5.9-11.6 µmol/L) to 54 mg/dL (1.6 µmol/L) (Fig. 3). As fresh-frozen plasma (FFP)-related allergies can worsen airway edema, we initially avoided FFP administration. Prior to the second DFPP, coagulation parameters were as follows: fibrinogen 134 mg/dL (4.0µmol/L), prothrombin international normalized ratio 1.15 (RR: 0.8-1.1), activated partial thromboplastin time 23.4 seconds (RR: 25-37 seconds), and Factor XIII 28% (RR: 70-140%). After the second session, 2 units of FFP were administered when fibrinogen levels dropped below 50 mg/dL (1.5 µmol/L). The third and fourth DFPP sessions were timed on days 19 and 24, respectively, to reduce the need for FFP and optimize preoperative coagulation. During this interval, FT3 and antibodies rebounded (Table 1). Four units of FFP were administered on day 26 during the preparation for thyroidectomy (Fig. 3).

**Table 1:** All laboratory results were obtained on the same day, immediately before and after each DFPP session

DFPP session	Day from presentation	Parameter	Before DFPP	After DFPP	Reference range
The first DFPP	Day 14	FT4 (ng/dL) [pmol/L]	6.60 ng/dL [84.96 pmol/L]	5.28 ng/dL [67.96 pmol/L]	0.70-1.48 ng/dL [9.01-19.05 pmol/L]
		FT3 (pg/mL) [pmol/L]	25.10 pg/mL [38.56 pmol/L]	18.30 pg/mL [28.11 pmol/L]	1.71-3.71 pg/mL [2.63-5.70 pmol/L]
		TRAb (IU/L)	309.00 IU/L	153.00 IU/L	< 1.22 IU/L
		TSAb (%)	3920%	1700%	< 130%
The second DFPP	Day 17	FT4 (ng/dL) [pmol/L]	4.21 ng/dL [54.19 pmol/L]	3.46 ng/dL [44.54 pmol/L]	0.70-1.48 ng/dL [9.01-19.05 pmol/L]
		FT3 (pg/mL) [pmol/L]	14.90 pg/mL [22.89 pmol/L]	13.00 pg/mL [19.97 pmol/L]	1.71-3.71 pg/mL [2.63-5.70 pmol/L]
		TRAb (IU/L)	198.00 IU/L	38.90 IU/L	< 1.22 IU/L
		TSAb (%)	2080%	1070%	< 130%
The third DFPP	Day 19	FT4 (ng/dL) [pmol/L]	2.77 ng/dL [35.66 pmol/L]	2.62 ng/dL [33.72 pmol/L]	0.70-1.48 ng/dL [9.01-19.05 pmol/L]
		FT3 (pg/mL) [pmol/L]	8.48 pg/mL [13.03 pmol/L]	8.64 pg/mL [13.27 pmol/L]	1.71-3.71 pg/mL [2.63-5.70 pmol/L]
		TRAb (IU/L)	111.00 IU/L	34.70 IU/L	< 1.22 IU/L
		TSAb (%)	1090%	820%	< 130%
The fourth DFPP	Day 24	FT4 (ng/dL) [pmol/L]	2.21 ng/dL [28.45 pmol/L]	2.31 ng/dL [29.73 pmol/L]	0.70-1.48 ng/dL [9.01-19.05 pmol/L]
		FT3 (pg/mL) [pmol/L]	10.9 pg/mL [16.74 pmol/L]	11.2 pg/mL [17.20 pmol/L]	1.71-3.71 pg/mL [2.63-5.70 pmol/L]
		TRAb (IU/L)	103.00 IU/L	29.40 IU/L	< 1.22 IU/L
		TSAb (%)	833%	562%	< 130%

Abbreviations: DFPP, double filtration plasmapheresis; FT3, free triiodothyronine; FT4, free thyroxine; TRAb, thyroid-stimulating hormone receptor antibody; TSAb, thyroid-stimulating antibody.

**Figure 4:**
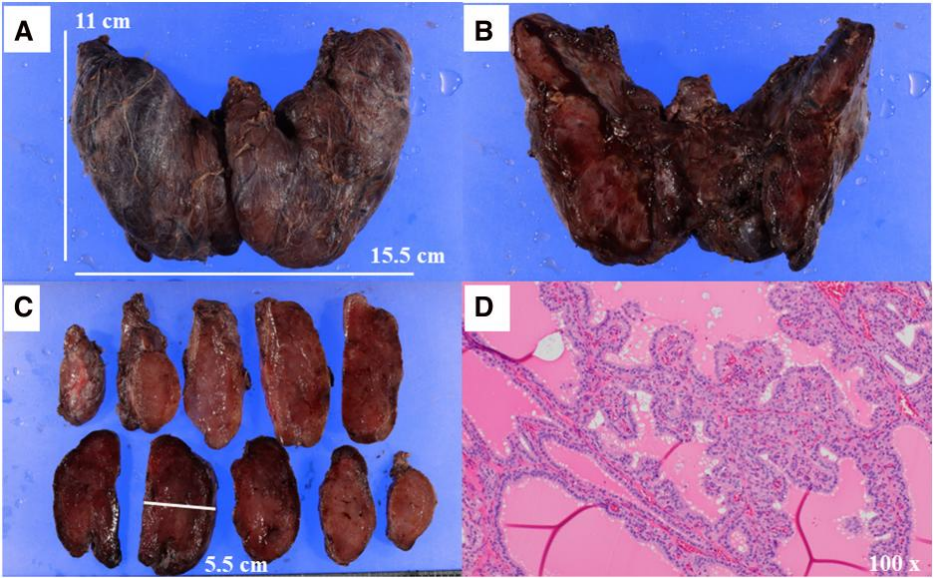
Pathological findings of yielded thyroid specimen. (A-C): The size of the thyroid gland was 15.5 cm × 11 cm × 5.5 cm. (D): The thyroid follicles were of unequal size, with partially enlarged follicular epithelial cells in a cuboidal to cylindrical shape and papillary proliferation in the lumen of the follicles. In addition, colloidal vacuole formation was observed in the vicinity of epithelial cells, a finding consistent with Graves disease.

Total thyroidectomy was performed 2 days after the fourth DFPP, with thyroid function tests showing improvement (FT4: 1.94 ng/dL (24.97 pmol/L), FT3: 8.47 pg/mL (13.1 pmol/L), TRAb: 35.4 IU/L; Table 1). Owing to the significant tracheal compression, extracorporeal membrane oxygenation (ECMO) was prepared as a precautionary measure; however, oral intubation was successfully performed without its use. The procedure yielded a thyroid specimen measuring 15.5 cm × 11 cm × 5.5 cm and weighing 405 g (Fig. 4A-4C), with blood loss of 359 mL. No blood transfusion was required during the surgery. Histopathological examination confirmed features consistent with GD (Fig. 4D).

## Outcome and follow-up

The postoperative course included hypothyroidism and transient hypocalcemia. She was discharged on postoperative day 13 with levothyroxine 125 mcg, calcium lactate 4 g, and alfacalcidol 4 mcg daily without complications.

At a month post-thyroidectomy, she demonstrated stable thyroid function under levothyroxine replacement therapy (125 mcg/day), with laboratory values showing FT4 1.61 ng/dL (20.72 pmol/L), FT3 2.45 pg/mL (3.76 pmol/L), and gradual reduction in autoantibody levels (TSAb: 280%, TRAb: 24.8 IU/L). Calcium homeostasis remained adequately controlled with calcium lactate 4 g and alfacalcidol 4 mcg supplementation, achieving a serum calcium level of 8.1 mg/dL (2.1 mmol/L) (RR: 8.4-10.2 mg/dL; 2.1-2.6 mmol/L). However, the persistently low intact parathyroid hormone level of 8.36 pg/mL (0.89 pmol/L) (RR: 22.4-88.2 pg/mL; 2.40-9.40 pmol/L) indicated ongoing parathyroid dysfunction.

## Discussion

This case highlights key aspects of ATD-resistant GD with a massive goiter. The patient acknowledged poor adherence to ATD therapy prior to admission, however, the persistence of hyperthyroidism despite inpatient therapy confirmed true ATD resistance. Airway compression necessitated urgent intervention; therefore, DFPP was performed as a bridge to surgery.

ATD resistance in this case can be explained by several mechanisms. MMI inhibits thyroid peroxidase (TPO) [4] and its efficacy depends on adequate intrathyroid concentrations [5]. Enlarged glands may require higher doses or longer treatment for sufficient TPO inhibition [6]. Additionally, elevated pretreatment thyroid hormone levels can reflect a large intrathyroid iodine pool [7], which interferes with MMI action and may promote oxidation [8]. Her dietary iodine intake was normal. While hormone levels were expectedly high in untreated GD, the extreme elevation suggested possible ATD resistance in this case. Alternative strategies were considered, including KI and high-dose glucocorticoids. High-dose glucocorticoids not only inhibit the synthesis and secretion of thyroid hormones but also suppress the peripheral from T4 to T3 [9]. Although these therapies produced modest improvement, they failed to achieve adequate control of the thyroid hormone levels.

Thus, the implementation of PE merits attention. Conventional PE can lower thyroid hormone levels in severe cases requiring urgent thyroidectomy [10], but often necessitates extensive FFP replacement, posing risks. DFPP selectively removes pathogenic high-molecular-weight substances while preserving albumin and coagulation factors [11], thereby reducing the need for plasma transfusion and the risk of transfusion-related reactions, which is particularly advantageous in the perioperative setting for patients with airway compromise. Management of coagulation parameters requires careful consideration. Despite normal coagulation times, we monitored Factor XIII levels, as its depletion can lead to bleeding complications that may not be detected by routine coagulation testing [12]. Previous reports have noted bleeding tendencies during surgery, even with normal screening tests performed 24 hours after PE [10]. Our strategy of spacing the final DFPP session 48 hours before surgery and administering FFP the day before the operation proved effective in preventing bleeding complications.

The temporary nature of DFPP effects was evident in our case, which is consistent with previous observations of conventional PE [13, 14]. The rebound in thyroid hormone levels and antibodies when DFPP sessions were spaced beyond 48 hours highlights the need for optimal timing.

Perioperative management poses airway challenges. ECMO standby has been reported for malignant goiters but is rarely used in benign disease [15]. Guidelines recommend euthyroid status before surgery [16], but surgery is feasible with suboptimal yet improved function if precautions are taken.

Notably, the correlation between thyroid volume and ATD resistance may inform treatment algorithms, potentially guiding early consideration of DFPP in similar cases [17]. Furthermore, DFPP effects may extend beyond antibody reduction to broader immune alterations, requiring investigation [18].

We conclude that GD patients with large goiters and markedly elevated pre-treatment hormone levels may be resistant to conventional therapy. DFPP can serve as an effective bridge to surgery with advantages over traditional PE, but timing is critical as effects are temporary and rebound may occur beyond 48 hours. Careful perioperative coagulation management is essential for safety.

## Learning points

- In GD with thyroid enlargement and elevated baseline thyroid hormone levels, resistance to ATD should be anticipated, prompting early consideration of adjunctive therapies.
- DFPP can be an effective bridge to surgery in cases of ATD-resistant thyrotoxicosis, offering advantages over conventional PE by selectively removing antibodies while preserving coagulation components.
- Proper DFPP timing is essential to maximize benefits and reduce perioperative risks. A 48-hour interval between the final DFPP and surgery, along with appropriate administration of FFP, can help reduce the risk of bleeding complications.
- Successful surgery for massive goiters requires comprehensive perioperative planning, including thyroid hormone optimization, coagulation management, and airway protection, even without full biochemical normalization.

## Data Availability

The original data generated and analyzed in this case report are included in this published article.

## Abbreviations

ATD: antithyroid drug
BP: blood pressure
DFPP: double filtration plasmapheresis
ECMO: extracorporeal membrane oxygenation
FFP: fresh-frozen plasma
GD: Graves disease
HR: heart rate
KI: potassium iodide
PE: plasma exchange
RR: reference range
TPO: thyroid peroxidase
TSH: thyroid-stimulating hormone
WHO: World Health Organization
